# Aerosol *Mycobacterium tuberculosis* Infection Causes Rapid Loss of Diversity in Gut Microbiota

**DOI:** 10.1371/journal.pone.0097048

**Published:** 2014-05-12

**Authors:** Kathryn Winglee, Emiley Eloe-Fadrosh, Shashank Gupta, Haidan Guo, Claire Fraser, William Bishai

**Affiliations:** 1 Department of Medicine, Johns Hopkins University, Baltimore, Maryland, United States of America; 2 Institute for Genome Sciences, University of Maryland, Baltimore, Maryland, United States of America; 3 Howard Hughes Medical Institute, Chevy Chase, Maryland, United States of America; Fundació Institut d'Investigació en Ciències de la Salut Germans Trias i Pujol. Universitat Autònoma de Barcelona. CIBERES, Spain

## Abstract

*Mycobacterium tuberculosis* is an important human pathogen, and yet diagnosis remains challenging. Little research has focused on the impact of *M. tuberculosis* on the gut microbiota, despite the significant immunological and homeostatic functions of the gastrointestinal tract. To determine the effect of *M. tuberculosis* infection on the gut microbiota, we followed mice from *M. tuberculosis* aerosol infection until death, using 16S rRNA sequencing. We saw a rapid change in the gut microbiota in response to infection, with all mice showing a loss and then recovery of microbial community diversity, and found that pre-infection samples clustered separately from post-infection samples, using ecological beta-diversity measures. The effect on the fecal microbiota was observed as rapidly as six days following lung infection. Analysis of additional mice infected by a different *M. tuberculosis* strain corroborated these results, together demonstrating that the mouse gut microbiota significantly changes with *M. tuberculosis* infection.

## Introduction

*Mycobacterium tuberculosis*, the causative agent of tuberculosis (TB), infects one third of the world's population, and caused 8.7 million new cases and 1.4 million deaths in 2010 alone [1]. Although the mouse immunopathologic response to TB differs from the response in humans, the mouse model is frequently used to study the virulence of different strains of *M. tuberculosis*, and to assess TB drug efficacy [2]. Upon aerosol infection, the bacteria promptly replicate in the lungs, and disseminated bacilli are observed in the spleen and liver two to four weeks later. Beginning at four weeks post-infection, the mouse adaptive immune system achieves partial control of the infection, resulting in a plateau in bacterial burden [3]. The mouse will develop granuloma-like lesions in the lung and, with a high infecting dose, will eventually succumb to the bacteria and die.

One aspect of TB infection that remains largely unexplored is the role of the resident microbiota (the microorganisms that collectively live on or in mammals). It is now well recognized that the gastrointestinal microbiota maintain a complex, reciprocal relationship with the host immune system [4], [5], [6], [7]. Moreover, differences in fecal microbiota composition and functional potential have been identified in individuals with various disease states when compared to healthy individuals, including inflammatory bowel disease, arthritis, type 2 diabetes, and asthma [8], [9], [10]. The gut microbiota also plays a prominent role in both enteric bacterial and viral infections [11], [12], [13], [14]. However, there have been few studies focused on changes in the gut microbiota in response to TB, especially in the absence of antibiotics.

To date, only one other study has focused on the gut microbiota during TB infection. Dubourg and colleagues utilized culture-dependent and -independent methods to evaluate bacterial and fungal diversity within a single patient at a single time, and found that there was an impoverished community dominated by only a few phylotypes [15]. However, this patient had been treated with broad-spectrum antibiotics for four months, which can greatly alter the gut microbiota. Another study concluded that there was a difference between the sputum microbial composition of TB patients and healthy controls [16]. Conversely, a different paper focused on human sputum samples found no difference between TB patients and healthy controls [17].

Given the interaction between the resident microbiota and the immune system, as well as competition among the microbiota and invading pathogens, we hypothesized that *M. tuberculosis* would cause a shift in the gut microbiota. Here, we present a longitudinal survey using 16S ribosomal gene sequencing of the gut microbiota in a mouse model for TB. We assessed bacterial community composition and diversity prior to infection with *M. tuberculosis* CDC1551 and throughout infection until death. Further, we evaluated the gut microbiota from additional mice infected by a different *M. tuberculosis* strain (H37Rv) for comparison to the longitudinal study.

## Results

### Compositional changes in the gut microbiota during *M. tuberculosis* infection

**Figure 1:**
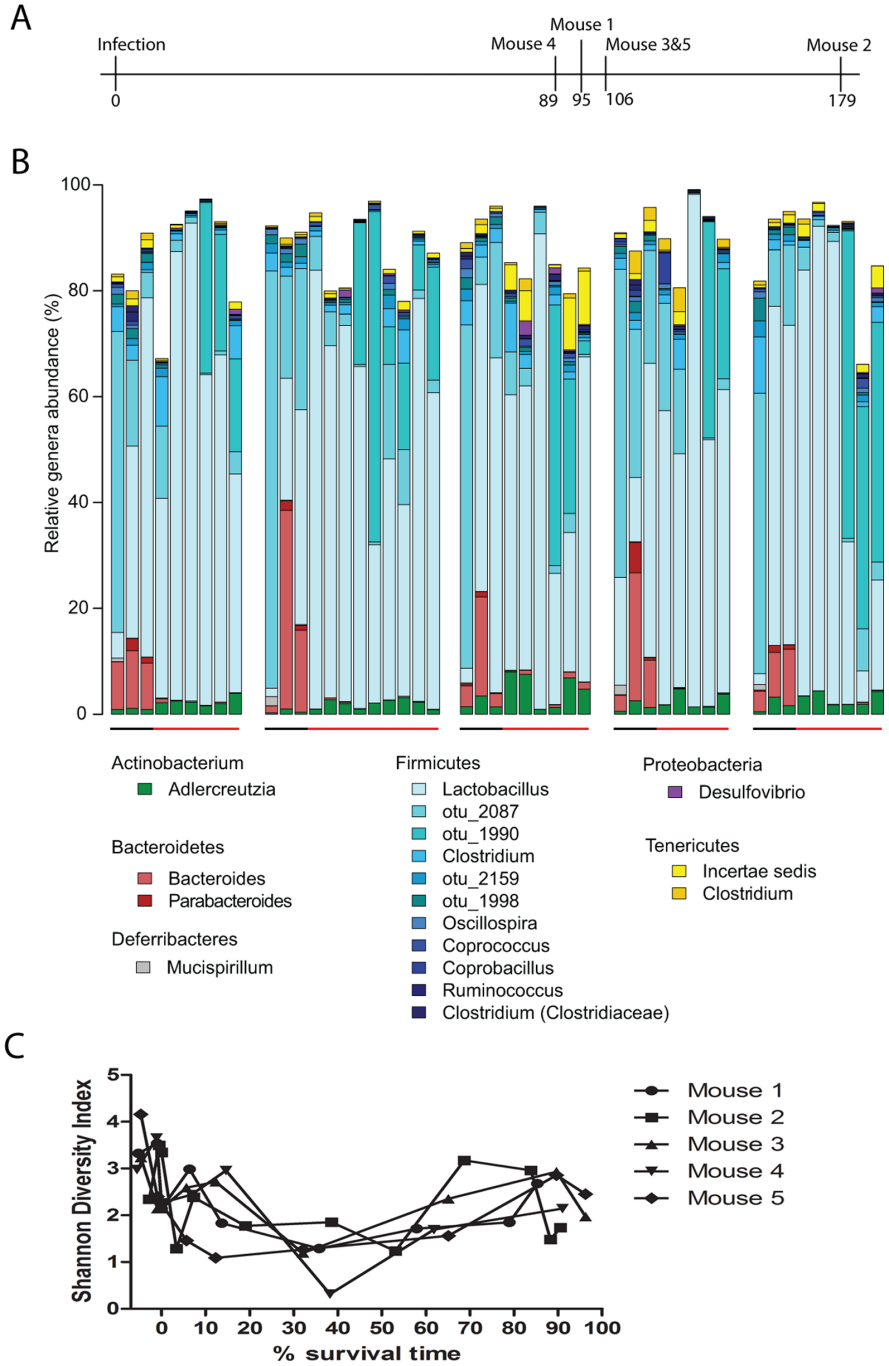
Community structure of individual *M. tuberculosis* CDC1551 infected mice over time. (**A**) Survival time in days post-infection for each mouse. (**B**) Phylogenetic profile of bacterial genera. Stacked bar charts in chronological order for each mouse of the 18 main genera identified based on ≥1% abundance present in at least two samples. Unclassified sequences are not shown. Black colored bars along x-axis indicate samples taken prior to infection, while red colored bars indicate post-infection. Each group represents an individual mouse, followed to death. The mice are represented sequentially, with mouse 1 on the left, and mouse 5 on the right. (**C**) Community diversity in each sample as measured by the Shannon diversity index, plotted against the percent survival time.

To study the impact of *M. tuberculosis* infection on the gut microbiota, we infected Balb/c mice with *M. tuberculosis* CDC1551, and monitored them until death. We collected fecal samples at time points prior to infection (pre-infection) and throughout infection (post-infection), selecting for analysis three pre-infection samples as controls, as well as samples from the first two weeks post-infection, the last two weeks prior to death and once per month in between (**Figure 1a** **, Table S1** and **Figure S1**). The fecal microbiota was characterized by 454 pyrosequencing of bacterial 16S rRNA gene amplicons (V1-V2 region) from the five infected mice. A total of 297,156 high-quality sequences were generated, corresponding to an average 6,322 reads per sample with an average length of 250 base pairs. These sequences were clustered into operational taxonomic units (OTUs) at 97% pairwise identity and taxonomically classified using the greengenes database [18]. The most abundant genera are shown in **Figure 1b**.

We further analyzed overall community diversity using the Shannon diversity index, a common ecological diversity measure, which takes into account both the number of species (OTUs) present and their relative abundance (**Figure 1c**). There was an initial decrease in diversity in all mice post-infection, followed by a recovery in diversity until death or one week prior to death. This was true even for mouse 2, which survived 73 days longer than any of the other mice. These trends were also observed using the Inverted Simpson diversity index, which takes into account community richness, abundance, and is less sensitive to rare OTUs compared to the Shannon diversity index (**Figure S2**).

### Gut community composition and structure differ based on infection status

**Figure 2:**
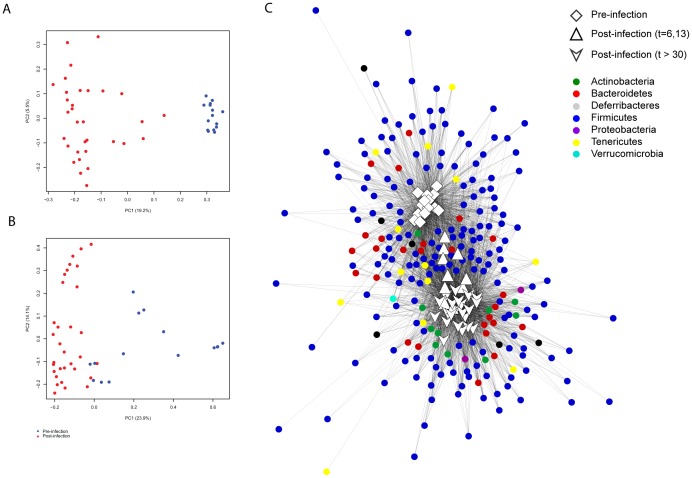
Composition of the gut microbiota significantly changes with *M. tuberculosis* CDC1551 infection. (**A**) Unweighted and (**B**) weighted Unifrac measures of beta-diversity visualized using Principle Coordinate Analysis (PCoA) following individual mice over time with *M. tuberculosis* CDC1551 infection. Blue dots indicate samples collected pre-infection. Red dots indicate samples collected post-infection. Variance for first two component axes is shown as percent of total variance. An analysis of molecular variance (AMOVA) was performed to test whether the separation of uninfected and TB-infected samples was statistically significant. In both unweighted and weighted Unifrac measures, there was a statistically significant difference (p<0.001). (**C**) Network analysis of OTUs partitioned among samples, using a five sequence cutoff, and colored by phylum.

To identify samples with similar microbial community structure and composition, we implemented multidimensional cluster analysis based on the weighted and unweighted UniFrac distances (**Figure 2a** and **Figure 2b**). UniFrac is a phylogenetically-aware measure of beta-diversity that can be used to compare OTU structure and community diversity. The weighted measure takes into account phylogenetic tree branch length. Both measures showed clear clustering among the uninfected samples taken pre-infection and the infected samples collected post-infection. We utilized an analysis of molecular variance (AMOVA), a statistical model similar to analysis of variance that is used to analyze differences in genetic diversity, to test whether the pre-infection and post-infection samples were statistically different, and found both weighted and unweighted UniFrac measures were significantly different (*p*<0.001). We further utilized a network analysis to evaluate how the OTUs were partitioned among samples, lending additional support for the separation of pre- vs. post-infection samples. Strikingly, the first two weeks after infection were found to be intermediate between the samples taken before infection and later in infection, with the Firmicutes distinguishing between the two conditions (**Figure 2c**).

**Figure 3:**
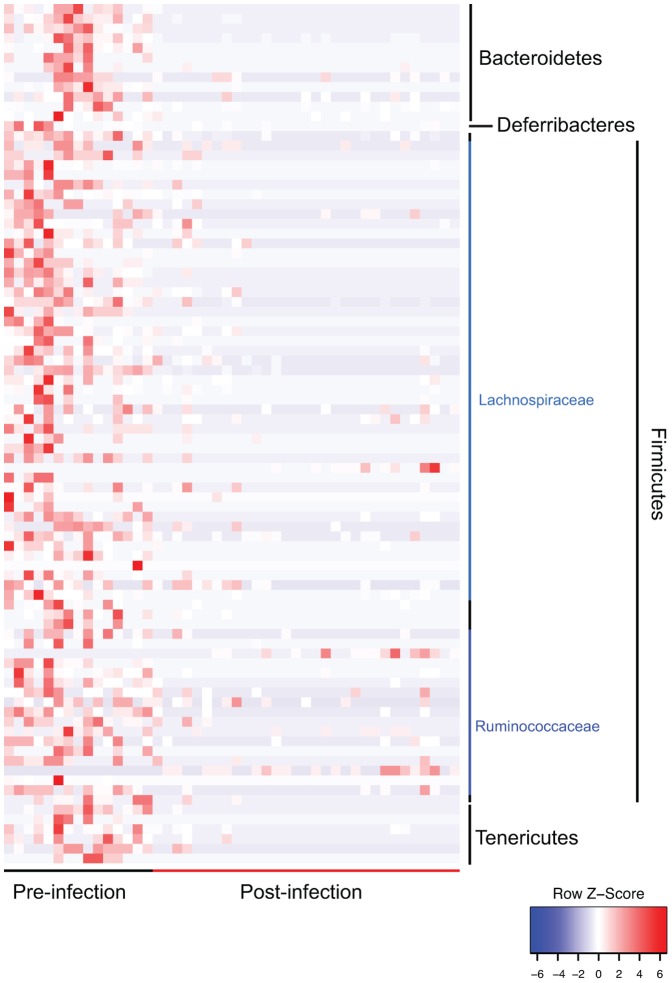
Differentially abundant OTUs identified between pre-infection and post-infection. OTUs are ordered by consensus taxonomic classification, with OTUs scaled by relative abundances for each row ranging from low relative abundance (blue) to high relative abundance (red).

We next implemented a categorical analysis to evaluate whether specific bacterial OTUs discriminated between pre-infection and the infected samples collected post-infection. Eighty-eight OTUs were found to be significantly differential (q<0.01); the majority belonged to the Firmicutes, specifically within the order Clostridiales (**Figure 3** **, Table S2**) [19]. The bulk of these OTUs classified within the Lachnospiraceae family, 31 affiliated with the unclassified Lachnospiraceae OTU2087 clade, and the Ruminococcaceae family. Interestingly, all of these significantly different OTUs were more abundant pre-infection compared to post-infection.

### Distinct changes in the gut community is independent of *M. tuberculosis* strain

**Figure 4:**
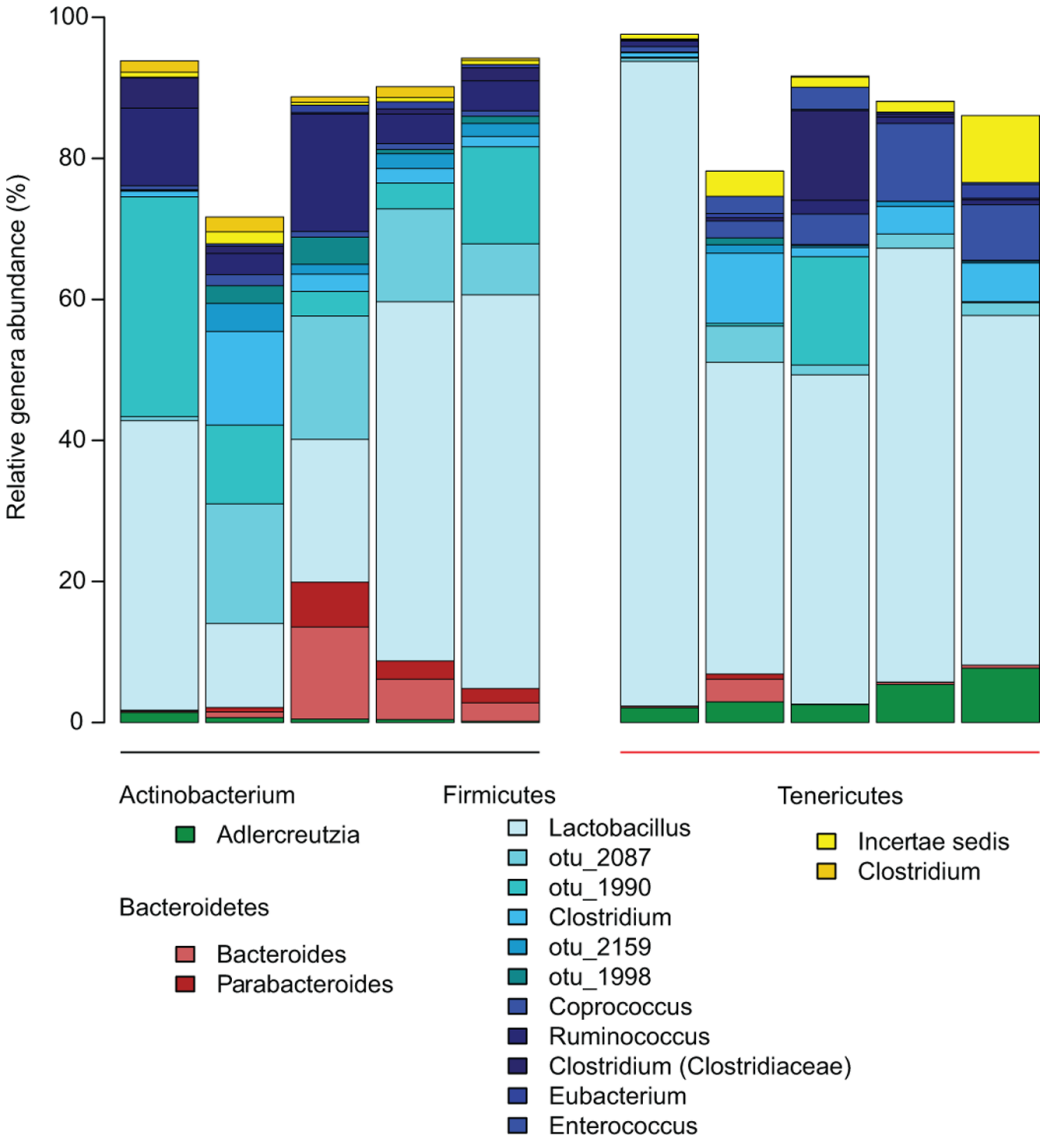
Phylogenetic profile of bacterial genera for uninfected and *M. tuberculosis* H37Rv infected mice. Stacked bar charts for uninfected and H37Rv-infected mice of the 16 main genera identified based on ≥1% abundance present in at least two samples. Unclassified sequences are not shown. The black colored bar along x-axis indicates the five uninfected mice, while the red colored bar indicates mice infected with H37Rv.

**Figure 5:**
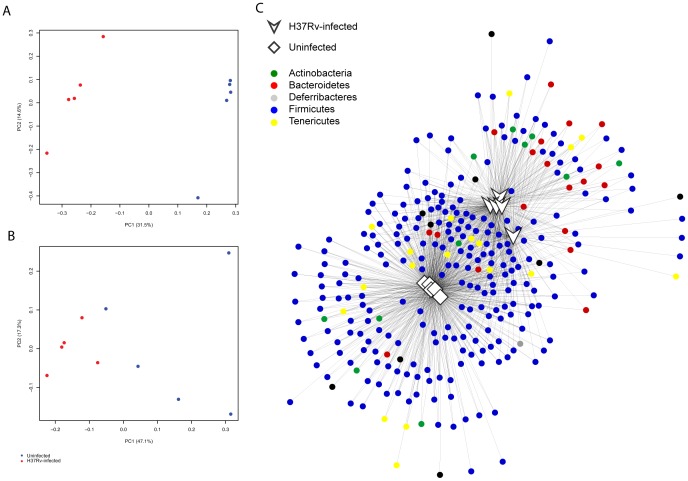
Gut microbiota composition of *M. tuberculosis* H37Rv infected mice is significantly different from uninfected mice. (**A**) Unweighted and (**B**) weighted Unifrac measures of beta-diversity visualized using Principle Coordinate Analysis (PCoA) for the comparison of H37Rv-infected mice to uninfected mice at a single time point. Blue dots indicate samples collected pre-infection. Red dots indicate samples collected post-infection. Variance for first two component axes is shown as percent of total variance. In both unweighted and weighted Unifrac measures, there was a statistically significant difference (AMOVA p≤0.005). (**C**) Network analysis of OTUs partitioned among samples, using a five sequence cutoff, and colored by phylum.

To determine if our previous results could be replicated with a different *M. tuberculosis* strain, we collected fecal samples from a single time point (46 days post infection) from *M. tuberculosis* H37Rv infected Balb/c mice and age-matched uninfected Balb/c mice that had been in the same facility for the same amount of time. Each group of mice was kept in two different cages to rule out variations in caging conditions. As described for the previous study, the fecal microbiota was characterized by 454 pyrosequencing of bacterial 16S rRNA gene amplicons (V1-V2 region) for this set of mice. A total of 148,466 high-quality sequences were generated, corresponding to an average 14,847 reads per sample, and were analyzed with the first experiment dataset to maintain consistency in OTU clustering (see Methods). The most abundant genera are shown in **Figure 4**. We implemented multidimensional cluster analysis based on the weighted and unweighted UniFrac distances and found distinct clustering between infected and uninfected samples, congruent with the results for the longitudinal study (**Figure 5a** **,** **Figure 5b**). Network analysis similarly confirmed these observations (**Figure 5c**). These results corroborate our earlier observations of distinct clustering between infected and uninfected animals.

**Figure 6:**
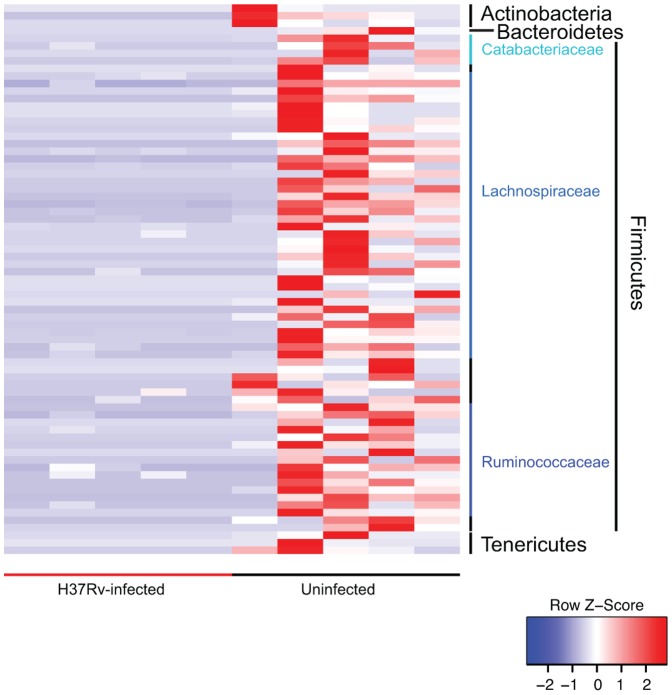
Differentially abundant OTUs identified between uninfected and *M. tuberculosis* H37Rv infected mice. OTUs are ordered by consensus taxonomic classification, with OTUs scaled by relative abundances for each row ranging from low relative abundance (blue) to high relative abundance (red).

Additionally, we found considerable overlap in the phylogenetic affiliation of the differentially abundant OTUs, which discriminated between uninfected and H37Rv-infected samples, compared to the CDC1551 infection longitudinal study. Seventy-three OTUs were found to be differential using the categorical analysis (q<0.01), the vast majority similarly within the Lachnospiraceae and Ruminococcaceae families (**Figure 6** **, Table S3**). Remarkably, the phylogenetic affiliations of the discriminatory OTUs mirrored those found in the CDC1551 infection longitudinal study and were highly abundant in the uninfected group, including OTU2087 (family Lachnospiraceae), OTU1995 (order Clostridiales), OTU1998 (genus *Catabacteriaceae*), OTU2159 (family Ruminococcaceae), OTU2166 (genus *Clostridium*), and OTU2176 (genus *Oscillospira*). However, while there was high congruence in the phylogenetic lineages observed between the two *M. tuberculosis* studies, only five OTUs specifically overlapped in both experiments (**Table S2 and Table S3)**.

## Discussion

We have monitored changes in the composition of the mouse gut microbiota from pre-infection to death, and shown that there is a clear difference between the microbial communities of infected and uninfected mice, results that have been confirmed by the use of two different strains of *M. tuberculosis*. These changes occur by day six post aerosol infection, indicating that this shift is very rapid. The prompt shift in the community after aerosol infection suggests that the gut microbiota is modulated during TB infection and may be responding to host immunological changes [20]. Furthermore, there is a consistent trend in these alterations, with a decrease in overall community diversity and discrete shifts in specific microbial taxa, despite the difference in survival time for the five mice studied. This trend occurred even in mouse 2, which survived 73 days longer than the other mice. Overall, we identified a number of OTUs significantly different between infected and uninfected mice, regardless of the infecting strain of *M. tuberculosis*. Mycobacterial DNA was not detected in any of these samples with the 16S rRNA pyrosequencing, but PCR of the *IS6110* insertion sequence, which is specific for *M. tuberculosis*, in the last sample collected prior to death identified TB in only three of the five mice (data not shown). We posit that the observed microbiota changes are not be mediated directly by the presence of *M. tuberculosis* in the gut, but instead represent crosstalk between the resident microbiota and the mucosal immune system. In fact, the minimum in diversity approximately corresponds to the time when the adaptive immune system begins to achieve a plateau in bacterial burden [21]. Thus, the loss of diversity could be a result of immune system activation, with a recovery once bacterial burden and immune activity have reached equilibrium.

We observed significant differences in the relative abundance of members within the Lachnospiraceae and Ruminococcaceae families (Clostridiales) in the two studies, with higher relative abundances in both the uninfected and pre-infected mice. It is known that some members of clusters IV and XIVa of the genus *Clostridium* (a genus in the *Clostridiales*) induce regulatory T cells [22]. Members of the Lachnospiracaceae have also been shown to decrease in abundance in inflammatory bowel disease (IBD) and colitis [23], [24]. Furthermore, a recent study found that a decrease in abundance of OTU 2087 was correlated with exacerbated asthma [25]. Future work to delineate immune-modulatory members of the Clostridiales, particularly those members associated with regulatory T cells and which have a role in *M. tuberculosis* immunity, will be critical.

While not as prominent as the differential *Clostridiales* OTUs, we additionally observed a subset of *Bacteroidales* OTUs differentially abundant in pre-infected and uninfected samples in the two studies. *Bacteroides* spp. have been shown to play important anti-inflammatory roles in inhibiting activation of the NF-κB pathway and induction of IL-10-producing T cells [6]. Furthermore, *Bacteroides fragilis* has been shown to modulate the T-helper type 1/2 (Th1/Th2) balance, another aspect of the immune system critical for the control of *M. tuberculosis* [26]. Further studies to characterize the relationship of specific *Bacteroides* species to the immune system are clearly needed.

Together, our results establish that the gut microbiota of mice changes significantly following aerosol infection by *M. tuberculosis*, and these differences may be related to the immune signaling from lung to gut. These changes begin as early as six days post infection and are characterized by an initial loss in diversity, which recovers to a significantly different composition. During this time, there are many alterations in the relative abundance of a number of OTUs, most significantly a decrease in members of the *Clostridiales* and *Bacteroidales*. These observations have important implications for our understanding of the interplay between the immune system and the gut microbiota. Further investigation into the interaction of the host immune system and the gut microbiota could lead to a more mechanistic understanding of gut immune functioning and the role the microbial fraction plays during TB infection.

## Materials and Methods

### Ethics Statement

This study was carried out in strict accordance with the recommendations in the Guide for the Care and Use of Laboratory Animals of the National Institutes of Health. The Animal Protocol MO11M120 was approved by the Institutional Animal Care and Use Committee of the Johns Hopkins University, and covers all animal procedures, including time-to-death. All efforts were made to minimize suffering.

### Bacterial Strains

*Mycobacterium tuberculosis* H37Rv and CDC1551 were routinely grown in Middlebrook 7H9 broth (Fisher Scientific, Waltham, MA) supplemented with 0.5% glycerol (Sigma, St. Louis, MO), 0.05% Tween 80 (Sigma) and 10% oleic acid-albumin-dextrose-catalase (OADC; Fisher Scientific) at 37°C with agitation.

### Animals

For the *M. tuberculosis* CDC1551 experiment, 4–6 week old female Balb/c mice were purchased from Charles River (Wilmington, MA). The five mice used for stool collection were toe tattooed for identification purposes and housed in the same cage. Mice were monitored daily as part of a time-to-death experiment and were provided with gel cups broken up feed biscuits when they became cachectic. For the *M. tuberculosis* H37Rv experiment, 19–20 gram female Balb/c mice were purchased from Charles River. Mice were housed in cages containing 5 animals. For each group, 3 mice were randomly selected from one cage, and 2 from another, for stool collection. For colony forming unit (CFU) counts of lungs and spleens, mice were euthanized by cervical dislocation.

### Stool Collection and Storage

For stool collection, each mouse was temporarily placed alone in a clean container until there was approximately 0.15 g stool in the container. The mouse was removed and the stool collected and stored in O-ring sealed tubes (Simport, Beloeil, Canada) at −80°C.

### Aerosol Infection

Mice were infected with log-phase broth cultures diluted in phosphate-buffered saline (PBS) using the Middlebrook inhalation exposure system (Glas-Col, Terre Haute, IN) in a single run. Bacterial burden was determined by sacrificing 3 mice per time point and enumerating CFUs on selective 7H11 plates (VWR, Radnor, PA).

### DNA Extraction and 16S rRNA Sequencing

Total DNA was extracted from 0.15 grams of stool using the ZR Fecal DNA isolation kit (ZYMO Research Corp., Irvine, CA) with modifications including an enzymatic pre-treatment with mutanolysin (5000 units ml^−1^, Sigma) and lysozyme (100 mg ml^−1^, Sigma) in conjunction with aggressive bead beating using 0.1 mm glass beads (BioSpec, Bartlesville, OK) and a bead-beater (BioSpec). Barcoded primers [27] were used to amplify the bacterial 16S rRNA gene region 27F-338R from 50 ng of purified DNA using AccuPrime High Fidelity DNA polymerase (Invitrogen, Grand Island, NY) in a total reaction volume of 25 µL. Reactions were run in a PTC-100 thermal controller (MJ Research, Waltham, MA) using the following cycling parameters: 5 min of denaturation at 95°C, followed by 25 cycles of 30 s at 95°C, 30 s at 55°C, and 60 s at 68°C, with a final extension at 72°C for 7 min. Amplicons were quantified using the Quant-iT PicoGreen dsDNA assay and equimolar amounts (100 ng) of the PCR product were mixed in a single tube. Amplification primers and reaction buffer were removed from each sample using the AMPure Kit (Agencourt, Brea, CA). The purified amplicon mixtures were sequenced by 454 FLX pyrosequencing using 454 Life Sciences primer A by the Genomics Resource Center at the Institute for Genome Sciences, University of Maryland School of Medicine.

### 16S rRNA sequence processing and analysis

Sequences generated from pyrosequencing of bacterial 16S rRNA gene amplicons from all mouse experimental groups were processed using mothur, version 1.27, according to the standard pipeline outlined in [28], [29]. Sequences were denoised, trimmed, quality and chimera checked using *de novo* uchime, and clustered into operational taxonomic units (OTUs) at 97% pairwise identity and aligned to the Silva reference alignment [30]. Taxonomic classifications were assigned using the naïve Bayesian classifier with the May 2011 release of the greengenes database [18]. Rarefied OTUs (randomly subsampled to normalize sequence counts) were used to calculate community diversity for each sample. Unweighted and weighted UniFrac distances, a phylogenetically-sensitive measure of beta-diversity, was used as input for the Principle Coordinate Analysis (PCoA), with visualizations performed using the R package vegan [31]. An analysis of molecular variance (AMOVA) was performed to test whether the groups were statistically significant. In addition, the program Metastats was used to identify differentially abundant OTUs between pre- and post-infection groups [19]. Network analysis to evaluate how the OTUs were partitioned among samples was performed using the make_otu_network.py script from QIIME [32] and visualized using Cytoscape [33].

### Nucleotide accession numbers

The 16S rRNA 454 pyrosequencing data has been deposited in the GenBank Sequence Read Archive under accession number SRA060942.

## Supporting Information

Figure S1 **Bacterial burden of** ***M. tuberculosis*** **CDC1551 infected mice.** *M. tuberculosis* colony forming units (CFUs) at day 0, 14, 28 and 56 in (**A**) the lungs and (**B**) the spleen of mice infected at the same time as the mice followed to death.
(TIF)

Figure S2 **Community diversity of** ***M. tuberculosis*** **CDC1551 infected mice.** Community diversity in each sample as measured by the Inverted Simpson diversity index, plotted against the percent survival time.
(TIF)

Table S1 **Time points assayed and time of death for each mouse.** Stool samples were collected every week post-infection. From these specimens, we selected samples from the first two weeks post-infection, the last two weeks prior to death and once per month in between for sequencing analysis. Time is given in days relative to infection.
(XLSX)

Table S2 **Differentially abundant OTUs in pre-infected samples and post-infected samples.** A q-value cutoff of q<0.01 was used.
(XLSX)

Table S3 **Differentially abundant OTUs in uninfected and infected samples.** A q-value cutoff of q<0.01 was used.
(XLSX)

## References

[1] World Health Organization. (2012) Tuberculosis. Available: http://www.who.int/mediacentre/factsheets/fs104/en/. Accessed: 28 Feb 2013.

[2] Nuermberger E (2008) Using animal models to develop new treatments for tuberculosis. Semin Respir Crit Care Med 29: 542–551. 18810687 10.1055/s-0028-1085705

[3] Flynn JL, Chan J (2001) Tuberculosis: latency and reactivation. Infect Immun 69: 4195–4201. 11401954 10.1128/IAI.69.7.4195-4201.2001 PMC98451

[4] Hooper LV, Littman DR, Macpherson AJ (2012) Interactions between the microbiota and the immune system. Science 336: 1268–1273. 22674334 10.1126/science.1223490 PMC4420145

[5] Jarchum I, Pamer EG (2011) Regulation of innate and adaptive immunity by the commensal microbiota. Curr Opin Immunol 23: 353–360. 21466955 10.1016/j.coi.2011.03.001 PMC3109238

[6] Maynard CL, Elson CO, Hatton RD, Weaver CT (2012) Reciprocal interactions of the intestinal microbiota and immune system. Nature 489: 231–241. 22972296 10.1038/nature11551 PMC4492337

[7] Nishio J, Honda K (2012) Immunoregulation by the gut microbiota. Cell Mol Life Sci 69: 3635–3650. 22527722 10.1007/s00018-012-0993-6 PMC11114866

[8] Penders J, Stobberingh EE, van den Brandt PA, Thijs C (2007) The role of the intestinal microbiota in the development of atopic disorders. Allergy 62: 1223–1236. 17711557 10.1111/j.1398-9995.2007.01462.x

[9] Qin J, Li Y, Cai Z, Li S, Zhu J, et al (2012) A metagenome-wide association study of gut microbiota in type 2 diabetes. Nature 490: 55–60. 23023125 10.1038/nature11450

[10] Yeoh N, Burton JP, Suppiah P, Reid G, Stebbings S (2013) The role of the microbiome in rheumatic diseases. Curr Rheumatol Rep 15: 314. 23378145 10.1007/s11926-012-0314-y

[11] Kuss SK, Best GT, Etheredge CA, Pruijssers AJ, Frierson JM, et al (2011) Intestinal microbiota promote enteric virus replication and systemic pathogenesis. Science 334: 249–252. 21998395 10.1126/science.1211057 PMC3222156

[12] Kamada N, Kim YG, Sham HP, Vallance BA, Puente JL, et al (2012) Regulated virulence controls the ability of a pathogen to compete with the gut microbiota. Science 336: 1325–1329. 22582016 10.1126/science.1222195 PMC3439148

[13] Sekirov I, Finlay BB (2009) The role of the intestinal microbiota in enteric infection. J Physiol 587: 4159–4167. 19491248 10.1113/jphysiol.2009.172742 PMC2754356

[14] Wilks J, Golovkina T (2012) Influence of microbiota on viral infections. PLoS Pathog 8: e1002681. 22615558 10.1371/journal.ppat.1002681 PMC3355081

[15] Dubourg G, Lagier JC, Armougom F, Robert C, Hamad I, et al.. (2013) The gut microbiota of a patient with resistant tuberculosis is more comprehensively studied by culturomics than by metagenomics. Eur J Clin Microbiol Infect Dis. 10.1007/s10096-012-1787-3 23291779

[16] Cui Z, Zhou Y, Li H, Zhang Y, Zhang S, et al (2012) Complex sputum microbial composition in patients with pulmonary tuberculosis. BMC Microbiol 12: 276. 23176186 10.1186/1471-2180-12-276 PMC3541192

[17] Cheung MK, Lam WY, Fung WY, Law PT, Au CH, et al (2013) Sputum Microbiota in Tuberculosis as Revealed by 16S rRNA Pyrosequencing. PLoS One 8: e54574. 23365674 10.1371/journal.pone.0054574 PMC3554703

[18] Wang Q, Garrity GM, Tiedje JM, Cole JR (2007) Naive Bayesian classifier for rapid assignment of rRNA sequences into the new bacterial taxonomy. Appl Environ Microbiol 73: 5261–5267. 17586664 10.1128/AEM.00062-07 PMC1950982

[19] White JR, Nagarajan N, Pop M (2009) Statistical methods for detecting differentially abundant features in clinical metagenomic samples. PLoS Comput Biol 5: e1000352. 19360128 10.1371/journal.pcbi.1000352 PMC2661018

[20] Gill N, Wlodarska M, Finlay BB (2010) The future of mucosal immunology: studying an integrated system-wide organ. Nat Immunol 11: 558–560. 20562837 10.1038/ni0710-558

[21] Chackerian AA, Alt JM, Perera TV, Dascher CC, Behar SM (2002) Dissemination of Mycobacterium tuberculosis is influenced by host factors and precedes the initiation of T-cell immunity. Infect Immun 70: 4501–4509. 12117962 10.1128/IAI.70.8.4501-4509.2002 PMC128141

[22] Atarashi K, Tanoue T, Shima T, Imaoka A, Kuwahara T, et al (2011) Induction of colonic regulatory T cells by indigenous Clostridium species. Science 331: 337–341. 21205640 10.1126/science.1198469 PMC3969237

[23] Costa MC, Arroyo LG, Allen-Vercoe E, Stampfli HR, Kim PT, et al (2012) Comparison of the fecal microbiota of healthy horses and horses with colitis by high throughput sequencing of the V3-V5 region of the 16S rRNA gene. PLoS One 7: e41484. 22859989 10.1371/journal.pone.0041484 PMC3409227

[24] Frank DN, St Amand AL, Feldman RA, Boedeker EC, Harpaz N, et al (2007) Molecular-phylogenetic characterization of microbial community imbalances in human inflammatory bowel diseases. Proc Natl Acad Sci U S A 104: 13780–13785. 17699621 10.1073/pnas.0706625104 PMC1959459

[25] Russell SL, Gold MJ, Hartmann M, Willing BP, Thorson L, et al (2012) Early life antibiotic-driven changes in microbiota enhance susceptibility to allergic asthma. EMBO Rep 13: 440–447. 22422004 10.1038/embor.2012.32 PMC3343350

[26] Mazmanian SK, Liu CH, Tzianabos AO, Kasper DL (2005) An immunomodulatory molecule of symbiotic bacteria directs maturation of the host immune system. Cell 122: 107–118. 16009137 10.1016/j.cell.2005.05.007

[27] Hamady M, Walker JJ, Harris JK, Gold NJ, Knight R (2008) Error-correcting barcoded primers for pyrosequencing hundreds of samples in multiplex. Nat Methods 5: 235–237. 18264105 10.1038/nmeth.1184 PMC3439997

[28] Schloss PD, Gevers D, Westcott SL (2011) Reducing the effects of PCR amplification and sequencing artifacts on 16S rRNA-based studies. PLoS One 6: e27310. 22194782 10.1371/journal.pone.0027310 PMC3237409

[29] Schloss PD, Westcott SL, Ryabin T, Hall JR, Hartmann M, et al (2009) Introducing mothur: open-source, platform-independent, community-supported software for describing and comparing microbial communities. Appl Environ Microbiol 75: 7537–7541. 19801464 10.1128/AEM.01541-09 PMC2786419

[30] Pruesse E, Quast C, Knittel K, Fuchs BM, Ludwig W, et al (2007) SILVA: a comprehensive online resource for quality checked and aligned ribosomal RNA sequence data compatible with ARB. Nucleic Acids Res 35: 7188–7196. 17947321 10.1093/nar/gkm864 PMC2175337

[31] Oksanen J, Blanchet FG, Kindt R, Legendre P, Minchin PR, et al. (2011) vegan: Community Ecology Package. R package version 20-2. Available: http://CRANR-projectorg/package=vegan. Accessed: 28 Feb 2013.

[32] Caporaso JG, Kuczynski J, Stombaugh J, Bittinger K, Bushman FD, et al (2010) QIIME allows analysis of high-throughput community sequencing data. Nat Methods 7: 335–336. 20383131 10.1038/nmeth.f.303 PMC3156573

[33] Smoot ME, Ono K, Ruscheinski J, Wang PL, Ideker T (2011) Cytoscape 2.8: new features for data integration and network visualization. Bioinformatics 27: 431–432. 21149340 10.1093/bioinformatics/btq675 PMC3031041

