# Supplementary figures and images for: Aerosol Mycobacterium tuberculosis Infection Causes Rapid Loss of Diversity in Gut Microbiota
Source: PLoS One. 2014 May 12;9(5):e97048. doi: 10.1371/journal.pone.0097048 (PMC4018338; doi:10.1371/journal.pone.0097048)

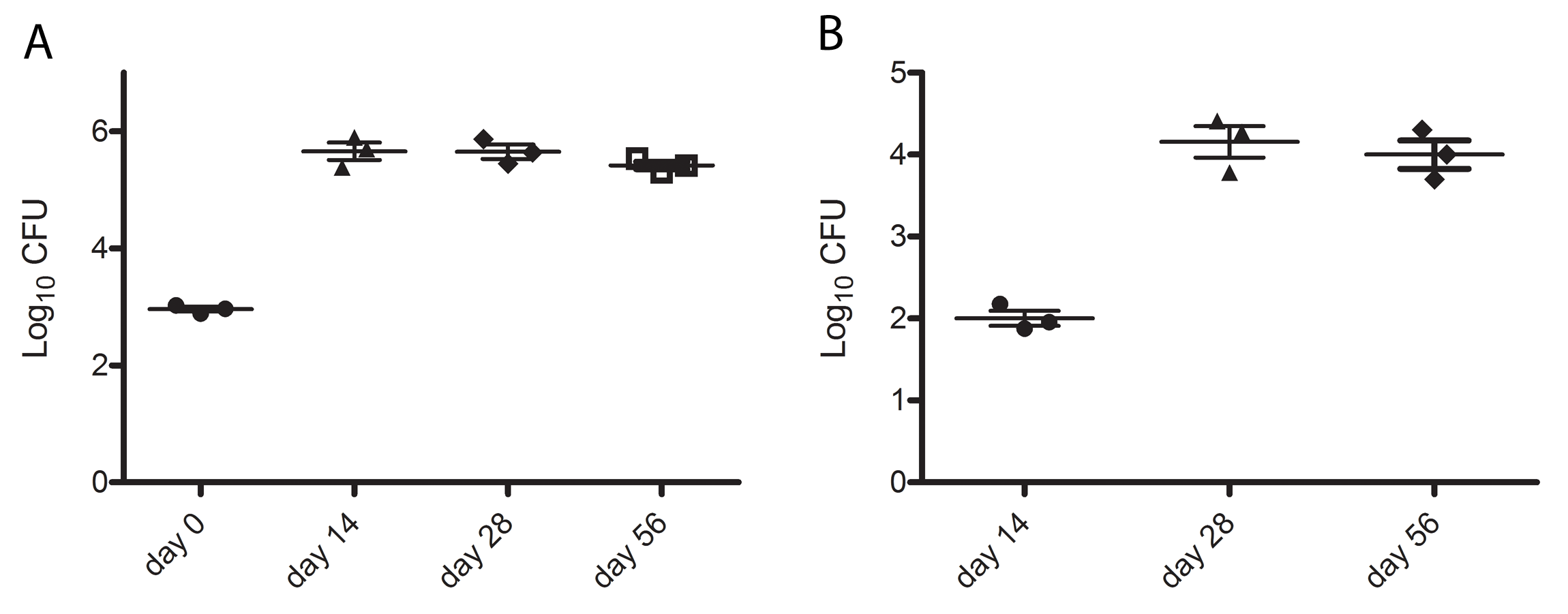

Supplement: Figure S1 — Bacterial burden of M. tuberculosis CDC1551 infected mice. M. tuberculosis colony forming units (CFUs) at day 0, 14, 28 and 56 in (A) the lungs and (B) the spleen of mice infected at the same time as the mice followed to death. (TIF) [file pone.0097048.s001.tif]

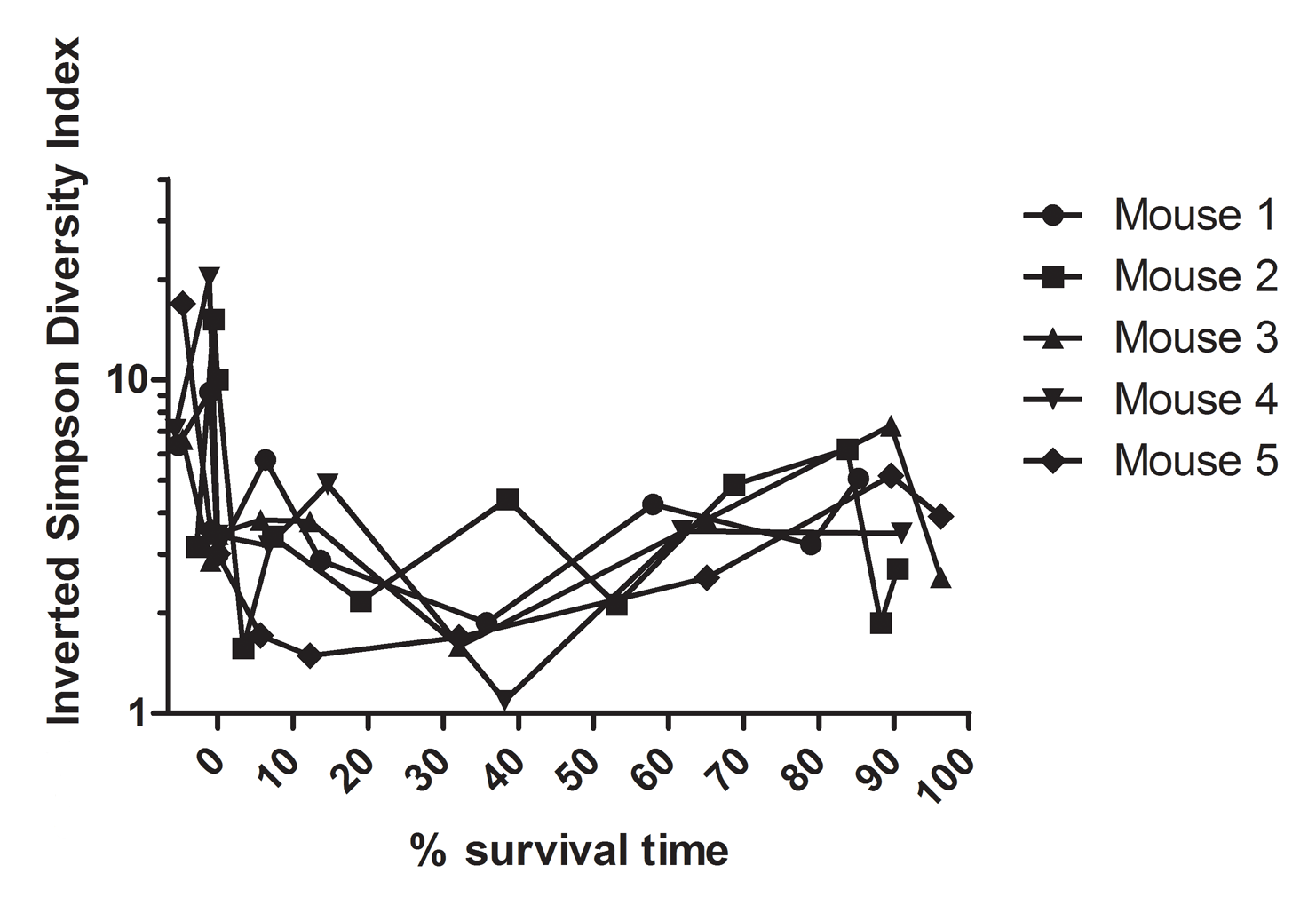

Supplement: Figure S2 — Community diversity of M. tuberculosis CDC1551 infected mice. Community diversity in each sample as measured by the Inverted Simpson diversity index, plotted against the percent survival time. (TIF) [file pone.0097048.s002.tif]
